# NK Cells Expressing the Inhibitory Killer Immunoglobulin-Like Receptors (iKIR) KIR2DL1, KIR2DL3 and KIR3DL1 Are Less Likely to Be CD16+ than Their iKIR Negative Counterparts

**DOI:** 10.1371/journal.pone.0164517

**Published:** 2016-10-12

**Authors:** Gamze Isitman, Alexandra Tremblay-McLean, Irene Lisovsky, Julie Bruneau, Bertrand Lebouché, Jean-Pierre Routy, Nicole F. Bernard

**Affiliations:** 1 Research Institute of the McGill University Health Centre (RI-MUHC), Montreal, Quebec, Canada; 2 Division of Experimental Medicine, McGill University, Montreal, Quebec, Canada; 3 Centre de Recherche du Centre Hospitalier de l’Université de Montréal (CRCHUM), Montreal, Quebec, Canada; 4 Department of Family Medicine, Université de Montréal, Montreal, Quebec, Canada; 5 Chronic Viral Illness Service, MUHC, Montreal, Quebec, Canada; 6 Department of Family Medicine, McGill University, Montreal, Quebec, Canada; 7 Division of Hematology, MUHC, Montreal, QC, Canada; 8 Division of Clinical Immunology, MUHC, Montreal, Quebec, Canada; McGill University AIDS Centre, CANADA

## Abstract

Natural Killer (NK) cell education, which requires the engagement of inhibitory NK cell receptors (iNKRs) by their ligands, is important for generating self-tolerant functional NK cells. While the potency of NK cell education is directly related to their functional potential upon stimulation with HLA null cells, the influence of NK cell education on the potency of the antibody dependent cellular cytotoxicity (ADCC) function of NK cells is unclear. ADCC occurs when the Fc portion of an immunoglobulin G antibody bridges the CD16 Fc receptor on NK cells and antigen on target cells, resulting in NK cell activation, cytotoxic granule release, and target cell lysis. We previously reported that education via the KIR3DL1/HLA-Bw4 iNKR/HLA ligand combination supported higher KIR3DL1^+^ than KIR3DL1^-^ NK cell activation levels but had no impact on ADCC potency measured as the frequency of granzyme B positive (%GrB^+^) targets generated in an ADCC GranToxiLux assay. A lower frequency of KIR3DL1^+^ compared to KIR3DL1^-^ NK cells were CD16^+^, which may in part explain the discrepancy between NK cell activation and target cell effects. Here, we investigated the frequency of CD16^+^ cells among NK cells expressing other iNKRs. We found that CD16^+^ cells were significantly more frequent among NK cells negative for the inhibitory KIR (iKIR) KIR2DL1, KIR2DL3, and KIR3DL1 than those positive for any one of these iKIR to the exclusion of the others, making iKIR^+^ NK cells poorer ADCC effectors than iKIR^-^ NK cells. The education status of these iKIR^+^ populations had no effect on the frequency of CD16^+^ cells.

## Introduction

Natural Killer (NK) cells acquire functional competence as they develop through a process known as education, which requires the interaction of inhibitory NK receptors (iNKRs) with their cognate human leukocyte antigen (HLA) ligands on neighboring cells [1–3]. Inhibitory NKRs include inhibitory Killer Immunoglobulin-like Receptors (iKIR), such as KIR2DL1 (2DL1), KIR2DL3 (2DL3), and KIR3DL1 (3DL1), as well as the C-type lectin receptor NKG2A. The 3DL1 receptor interacts with a subset of HLA-A and–B antigens that belong to the Bw4 subset [4,5]. Bw4 antigens differ from the remaining Bw6 HLA-B variants, which do not interact with 3DL1, at amino acids 77–83 of the HLA heavy chain [6]. Thus, NK cells from *Bw6* homozygotes with no HLA-A *Bw4* alleles can serve as controls for the effect of education though 3DL1 on NK cell function. The 2DL3 receptor interacts with HLA-C group 1 (C1) variants having an asparagine at position 80 of the heavy chain [7,8]. Other HLA-C variants with a lysine at this position belong to the C2 group and are ligands for 2DL1 [8]. The 2DL3 receptor can also bind certain allelic variants of C2, though with lower affinity than 2DL1 [9]. Therefore, 2DL3^+^ NK cells from individuals expressing the C1 ligand are educated, but are either uneducated or less potently educated in individuals expressing only C2 ligands. NKG2A interacts with non-classical major histocompatibility complex class I (MHC-I) HLA-E molecules that present leader peptides from many MHC-I proteins and certain viral derived epitopes [10–13]. NKG2A and HLA-E molecules are highly conserved and their effect on NK cell education is similar from one person to another [14].

NK cell education is a dynamic process whereby functionality can be tuned by the number of iNKRs engaged, the strength of interactions between iNKRs and their ligands and the potential additional engagement of activating NK cell receptors (aNKRs) [9,15–19]. The potency of NK cell education is related to the frequency of NK cells that are activated by stimuli such as HLA null cells and antibody (Ab) dependent NK cell stimulation by recombinant gp120 coated CEM.NKr-CCR5 (CEM) cells in the presence of anti-human immunodeficiency virus (HIV) Envelope (Env) specific Abs [16,18,20–23]. Previous work by our group noted inter-individual variation in NK cell mediated ADCC activity in a flow cytometry based ADCC GranToxiLux (ADCC-GTL) assay, which measured the %GrB^+^ target cells generated in this assay [24,25]. The target cells used in this assay were gp120 coated CEM cells and the source of Ab was HIVIG, immunoglobulin G (IgG) from pooled plasma from HIV infected subjects. We showed that this variability was not due to differences in the ability of 3DL1/HLA-B pairs to educate NK cells [24]. This finding was despite the observation that 3DL1^+^ NK cells that were educated through 3DL1/Bw4 interactions were stimulated by the same gp120 coated CEM target cells in an Ab dependent fashion to higher levels than 3DL1^-^ NK cells [22,24,26]. Furthermore, we showed that among 3DL1^+^ NK cells, there was a significantly lower frequency of CD16^+^ cells than among 3DL1^-^ NK cells. As CD16 is crucial to ADCC activity, this would compromise the ability of educated 3DL1^+^ NK cells to act as effector cells in terms of delivering GrB to target cells [24,26].

In this report we expanded on these results by investigating the proportion of CD16^+^ cells among NK cells expressing NKG2A, 2DL1, 2DL3, 3DL1, or none of these iNKR by adopting a gating strategy that focused on NK cells expressing one of these iNKR to the exclusion of the others stained for. We found NK cells exclusively expressing a single iKIR co-expressed CD16 less frequently than did NK cells expressing none of these iNKRs. On the other hand, NK cells exclusively expressing NKG2A only, co-expressed CD16 more frequently than did NK cells negative for these iNKRs. Furthermore, the frequency of CD16 on these single iKIR positive NK cell populations did not differ based on whether or not the NK cells developed in an environment that would have supported their education.

## Materials and Methods

### Ethics statement and study population

This study was conducted in accordance with the principles expressed in the Declaration of Helsinki. It was approved by the Institutional Review Boards of the Comité d’Éthique du Centre de Recherche du Centre Hospitalier de l’Université de Montréal and the Research Ethics Committee of the McGill University Health Centre—Montreal General Hospital. All individuals provided written informed consent for the collection of samples and subsequent analysis.

We studied 26 HIV seronegative donors. The MHC-I HLA-A, -B, and–C type of each study participants is shown in Table 1. Also shown in this table is the generic *3DL1/S1* genotype, whether a *2DL1* locus was present, whether an allele belonging the *2DL3* group encoded at the *2DL2/L3* locus was present, whether subjects carried a *Bw4* allele at either the *HLA-A* or–*B* locus, and whether they carried *HLA-C* alleles encoding antigens belonging to the C1 or C2 group or both.

**Table 1:** Study population HLA and KIR genotypes.

Donor	3DL1/S1^1^	2DL1^2^	2DL3	HLA-A	HLA-B	HLA-C	Bw4	C1/C2
1	3DL1	1	1	01:01, 03:01	44:03, 49	07:01, 16:01	1	C1
2	3DL1	1	1	24:01, 26:01	15:01, 57:01	05:01, 06:02	1	C2
3	HTZ	1	0	03:01, 32:01	13:02, 53:01	04:01, 06:02	1	C2
4	3DL1	1	1	01:01, 31:01	49:01	07:01	1	C1
5	3DL1	1	1	02:01, 03:01	07:02, 27:05	01:02, 07:02	1	C1
6	3DS1	1	1	02:01, 24:02	38:01, 78:01	07:02	1	C1
7	3DL1	1	1	02:01	07:02, 57:01	05:01, 06:02	1	C2
8	HTZ	1	1	03:01, 11:01	40, 57:01	02:02, 04:01	1	C2
9	3DL1	1	1	01:01, 23:01	14:02, 38:01	08:02, 12:02	1	C1
10	HTZ	1	0	02:01, 29:02	07:02, 44:03	07:02,16:01	1	C1
11	3DS1	1	1	02:01, 03:01	27:05, 47:01	02:02, 06:02	1	C2
12	HTZ	1	1	02:01, 03:01	07:02, 50:01	07:02, 16:02	0	C1
13	3DL1	1	1	01:01, 02:01	08:01, 40:01	03:02, 07:01	0	C1
14	3DL1	1	1	02:01	07:02, 08:01	07:01, 07:02	0	C1
15	3DL1	1	1	02:01, 30:02	07:02, 35:01	04:01, 07:02	0	C1/C2
16	3DL1	1	1	03:01, 11:01	07:02, 35:01	04:01, 07:02	0	C1/C2
17	3DL1	1	1	02:01, 24:02	44:02, 01:01	05:01, 08:02	1	C1/C2
18	3DL1	1	1	01:01, 26:01	38:01, 57:01	06:02, 12:03	1	C1/C2
19	HTZ	1	1	01:01, 23:01	44:03, 57:01	04:01, 06:02	1	C2
20	HTZ	1	1	11:01, 24:02	27:02, 53:03	02:02, 04:01	1	C2
21	HTZ	1	0	02:01, 11:01	35:01, 40:02	02:02, 04:01	0	C2
22	3DL1	1	1	01:01, 68:02	18:01, 57:01	05:01, 06:02	1	C1/C2
23	3DL1	1	1	02:01, 03:01	35:01, 40:01	03:04, 04:01	0	C1/C2
24	3DL1	1	1	02:01, 03:01	07:02, 08:01	07:01, 16:01	0	C1
25	3DL1	1	1	01:01, 24:02	51:01, 73:01	04:01, 07:01	1	C1/C2
26	3DL1	1	1	02:01, 25:01	18:01, 55:01	03:03, 12:03	0	C1

^1^ *KIR3DL1* generic genotype, 3DL1 = *KIR3DL1* homozygote, HTZ = *KIR3DL1/S1* heterozygote, 3DS1 = *KIR3DS1* homozygotes.

^2^ 0 = not present; 1 = present.

### Genotyping

*MHC-I* alleles were typed using commercial reagents (Atria Genetics Inc., South San Francisco, CA). Genotyping and allotyping of *3DL1/S1* was performed as previously described [27,28]. Presence of *2DL1* and *2DL2*/*2DL3* loci and alleles belonging to the *2DL3* group was determined using a KIR genotyping kit (One Lambda Inc., Canoga Park, CA). Presence of *2DL1*, *2DL2*, and *2DL3* alleles was verified by PCR using specific primers and conditions described by Kulkarni et al.[29]

### Cells

Peripheral blood mononuclear cells (PBMC) were isolated by density gradient centrifugation (Lymphocyte Separation Medium, Wisent, St. Jean Baptiste, QC, Canada) from whole blood obtained by venipuncture into tubes containing EDTA anticoagulant or by leukapheresis, as previously described [18,30]. The cells were then cryopreserved in 10% DMSO, with 90% fetal bovine serum (FBS). After thawing, cells were rested overnight in RPMI media containing 2 mM L-glutamine; 100 IU/ml penicillin; 100 μg/ml streptomycin; 10% FBS (R10) (all from Wisent) at 37°C in a 5% humidified CO_2_ incubator at a concentration of 1x10^6^ cells/ml.

### CD16 surface staining

Rested, unstimulated PBMCs were cell surface stained to identify the frequency of CD16^+^ expression among CD56^total/dim/bright^ and iNKR^+/-^ NK cell populations. Following staining with UV Live/Dead Fixable Stain (Invitrogen, Burlington, ON, Canada), surface staining was performed in the dark at room temperature on 1x10^6^ PBMCs per individual with Abs of the following specificities: CD3-BV785 (OKT3), CD56-BV711 (NCAM16.2), 3DL1-BV421 (DX9), CD57-FITC (HCD57), (all from BioLegend, San Diego, CA), NKG2A-PeCy7 (Z199, Beckman Coulter, Mississauga, ON, Canada), CD16-APC-Cy7 (3GB, BD Biosciences, Mississauga, ON, Canada), 2DL1-AlexaFluor700 (143211) and 2DL3-PE (180701; both from R&D Systems, Minneapolis, MN). Stained cells were then washed and fixed with 1% paraformaldehyde solution (PFA, Santa Cruz, Biotechnology, Inc, Dallas, TX). Between 300,000 and 400,000 events per sample were acquired using an LSRFortessa flow cytometer (BD). Data analysis was performed using FlowJo software v10 (Treestar, Ashland OR). Unstained, single color, and fluorescence minus one controls were used for each subject for multi-color compensation and gating purposes.

### Statistical analysis

Statistical analysis and graphical presentation were performed using GraphPad Prism 6 (GraphPad Software, Inc., La Jolla, CA). Wilcoxon matched pairs and Mann-Whitney tests were used to assess the significance of differences on 2 matched or unmatched groups, respectively. Friedman tests with Dunn’s post-test comparisons were used to assess the significance of more than 2 matched groups. Results were reported as medians and inter-quartile ranges (IQR). P-values less than 0.05 were considered significant.

## Results

### CD16, NKG2A, and CD57 expression in CD56^+^ NK cell populations

**Fig 1:**
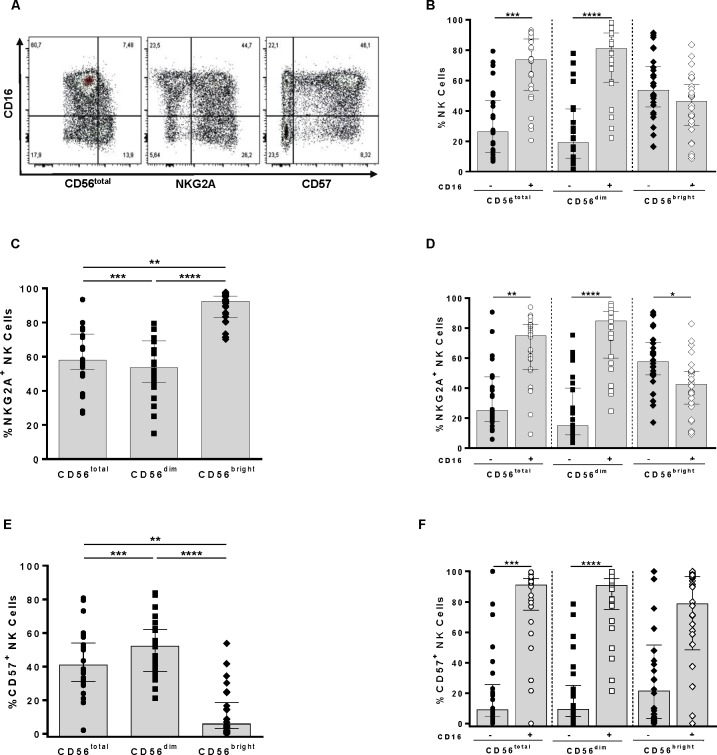
Frequency of CD16^+^, NKG2A^+^ and CD57^+^ NK cells and their subsets expressing or not CD16. **(A)** Representative gating strategy for determining the frequency of CD16^+/-^ cells on CD56^total^, CD56^dim^, and CD56^bright^ NK cell populations and on the NKG2A^-/+^ and CD57^-/+^ subsets of these populations. **(B)** Frequencies of CD16^+/-^ cells in the CD56^total^, CD56^dim^, and CD56^bright^ NK cell populations. Frequencies of expression of NKG2A **(C)** and CD57 **(E)** on CD56^total^, CD56^dim^, and CD56^bright^ NK cells. A Friedman test was used to assess the significance of matched between group differences. Frequencies of CD16^+^ and CD16^-^ cells within the NKG2A^+^ **(D)** and CD57^+^ **(F)** CD56^total^, CD56^dim^, and CD56^bright^ NK cell populations. Each data point represents results for 1 of 26 separate individuals. Bar height and error bars represent the median and interquartile range for the data set. Wilcoxon tests were used to determine significance of within subject differences for the indicated NK subsets linked by a line connecting the data sets. Significant values are shown; “*” = p< 0.05; “**” = p< 0.01; “***” = p< 0.001; “****” = p< 0.0001.

NK cells were defined as CD3^-^CD56^+^ cells from the live singlet lymphocyte gate. Two NK cell subsets were identified, i.e. the less mature CD3^-^CD56^bright^ cells, which represent approximately 10% of circulating NK cells, and the more differentiated CD3^-^CD56^dim^ population, which comprises the remainder of circulating NK cells [31–34] (S1A Fig). The frequencies of CD16^+^, CD16^+^NKG2A^+^, and CD16^+^CD57^+^ NK cells and their CD16^-^ counterparts within these three NK cell populations were assessed using the gating strategy shown in Fig 1A.

Consistent with observations made by others, the CD56^+^ and CD56^dim^ NK populations had higher frequencies of CD16^+^ than CD16^-^ cells (Fig 1B, left and middle panels and S1 Table), while the opposite was seen for CD56^bright^ NK cells (right panel) (p = 0.016, p = 0.005, and p = 0.043, respectively, Wilcoxon tests) [33]. NKG2A is an iNKR whose expression declines as NK cells mature [35,36]. The proportion of NKG2A^+^ NK cells was lower in total and CD56^dim^ than in the CD56^bright^ population (Fig 1C and S2 Table; p = 0.014 and p<0.0001, respectively, Dunn’s post-tests). Within the total CD56^+^ and CD56^dim^ NKG2A^+^ populations, there was a higher proportion of CD16^+^ than CD16^-^ cells, while there was a lower frequency of CD16^+^ than CD16^-^ cells within the NKG2A^+^CD56^bright^ population (Fig 1D, and S3 Table p = 0.043, p = 0.002, and p = 0.043, respectively, Wilcoxon).

CD57 is a marker of senescence and terminal differentiation [36–38]. In accordance with this, the frequency of CD57^+^ total CD56^+^ and CD56^dim^ NK cells was higher than that of CD57^+^CD56^bright^ NK cells (Fig 1E and S4 Table, p = 0.014 and p<0.0001, respectively, Dunn’s post-tests). A higher proportion of CD57^+^ total CD56^+^, CD56^dim^, and CD56^bright^ NK cells were CD16^+^ than CD16^-^, though the difference for CD57^+^CD56^bright^ NK cells did not achieve statistical significance, likely due to the low frequency of CD57^+^NK cells that were CD56^bright^ (Fig 1F and S5 Table, p = 0.01, p = 0.0002, and p = 0.4, respectively, Wilcoxon).

### CD16 and KIR expression on total CD56^+^ and CD56^dim^ NK cells

**Fig 2:**
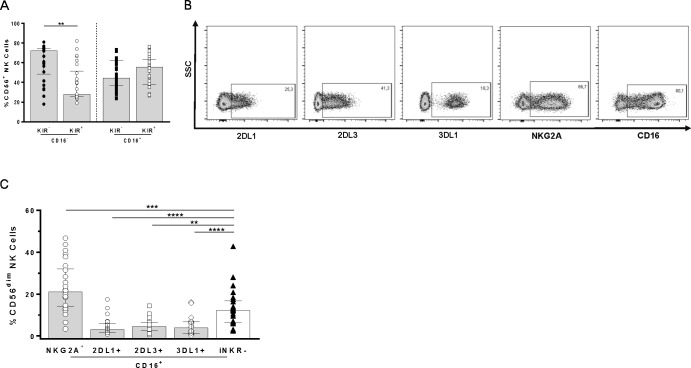
CD16 expression on total CD56^+^ NK cells populations expressing all combinations of iKIRs and on CD56^dim^ single iNKR expressing NK cell populations. **(A)** The frequency of KIR^+^ (expressing any Boolean combination of KIR2DL1 (2DL1), KIR2DL3 (2DL3), or KIR3DL1 (3DL1]) subsets in CD16^-^ and CD16^+^ total CD56^+^ NK cell populations. **(B)** The gating strategy for assessing the frequencies of single NKG2A, 2DL1, 2DL3, and 3DL1 and CD16 positive CD56^dim^ NK cells. **(C)** Frequencies of CD16 positive CD56^dim^ NK cells that are single positive for NKG2A (n = 26), 2DL1 (n = 25),2DL3 (n = 22), and 3DL1 (n = 22) or negative for all iNKR tested (iNKR^-^). Wilcoxon tests were used to determine significance of within subject differences for the indicated NK subsets. Each data point represents results a separate individual. Bar height and error bars represent the median and interquartile range for the data set. Significant values are shown; “**” = p< 0.01; “***” = p< 0.001; “****” = p< 0.0001.

We next assessed the frequency of total CD56^+^ NK cells that were positive or negative for CD16 and that expressed any combination of the iKIR 2DL1, 2DL3, and 3DL1 or none of these IKIR (Fig 2A and S5 Table). The proportion of CD16^-^ NK cells that expressed at least one of these iKIR was lower than those that expressed none. The frequency of CD16^+^ NK cells that co-expressed or not at least one of these iKIR did not differ significantly.

To further investigate the relationship between iNKR and CD16 expression, we adopted an exclusive gating strategy in order to examine the frequency of CD16^+^ cells among NK cells expressing NKG2A, 2DL1, 2DL3, or 3DL1 to the exclusion of the other iNKR and compared this frequency to that of CD16^+^ NK cells negative for all of these iNKR. For this analysis we used Boolean gating to measure the frequency of NKG2A^+^, 2DL1^+^, 2DL3^+^, 3DL1^+^, and CD16^+^ cells within the CD56^dim^ NK cell gate (Fig 2B and S6 Table). A higher frequency of CD56^dim^ NKs expressing NKG2A to the exclusion of the other iNKRs tested (NKG2A^+^2DL1^-^2DL3^-^3DL1^-^) co-expressed CD16, compared to cells negative for all four iNKRs (Fig 2C and S7 Table, 21.1 [15.5, 30.7] and 12.4 [6.4, 5.75], respectively, p = 0.0004 Wilcoxon). We found that a significantly lower proportion of cells expressing each of the three iKIR to the exclusion of the other iNKR (NKG2A^-^2DL1^+^2DL3^-^3DL1^-^, NKG2A^-^2DL1^-^2DL3^+^3DL1^-^, or NKG2A^-^2DL1^-^2DL3^-^3DL1^+^) were CD16^+^ compared to NK cells negative for all four iNKRs tested (Fig 2C and S7 Table, [3.1 (1.6, 5.88)], p<0.0001, [4.41 (2.8, 7.46)], p = 0.008, and [3.84 (1.56, 6.6)], p<0.0001 for comparisons with 2DL1, 2DL3 and 3DL1, respectively Wilcoxon). Together, these results show that NK cells expressing 2DL1, 2DL3, or 3DL1 alone are less likely to be CD16^+^ cells than iNKR^-^ NK cells. Additionally, CD56^dim^ NK cells that do not express the KIR studied, but that express NKG2A alone are more likely to co-express CD16 than iNKR^-^ cells.

### The effect of KIR education on CD16 Expression

**Fig 3:**
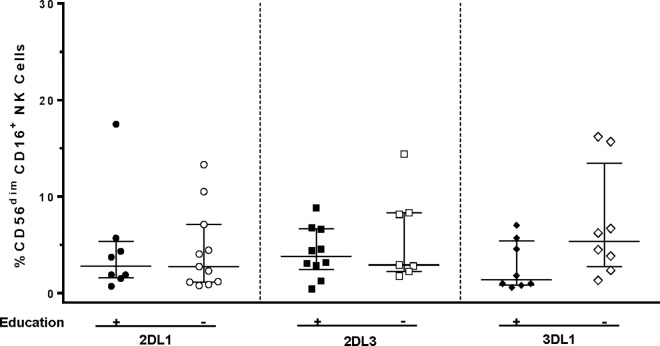
The frequency of educated and uneducated CD56^dim^ NK cell populations expressing CD16 and one of the inhibitory KIR KIR2DL1, KIR2DL3, or KIR3DL1. The frequency of CD16^+^ cells among educated KIR2DL1^+^ (2DL1) NKG2A^-^CD56^dim^ NK cells from *HLA-C2* homozygotes (n = 8) versus uneducated *HLA-C1* homozygotes (n = 11), educated KIR2DL3^+^ (2DL3) NKG2A^-^CD56^dim^ NK cells from *HLA-C1* homozygotes (n = 10) versus uneducated *HLA-C2* homozygotes (n = 7), and educated KIR3DL1^+^ (3DL1) NKG2A^-^CD56^dim^ NK cells from *Bw4* carriers (n = 8) versus uneducated *Bw6* homozygotes (n = 8). The lines and error bars through the datasets represent medians and interquartile ranges. Mann-Whitney tests assessing the significance of differences in the frequency of CD16^+^, single KIR positive cells in educated versus uneducated NK cell subsets found no significant differences.

CD56^dim^NKG2A^-^ NK cells expressing 2DL1, 2DL3, or 3DL1 were stratified according to whether they came from a study subject co-carrying or not a ligand for that KIR, which would support NK cell education through each receptor. We then compared the frequency of CD16^+^ CD56^dim^ NK cells from subjects whose KIR/HLA genotype would support versus not support education through each receptor (Fig 3 and S8 Table). Educated 2DL1^+^ cells from *HLA-C2* carriers were compared to uneducated 2DL1^+^ NK cells from *HLA-C1* homozygotes. Likewise, 2DL3^+^ cells from *HLA-C1* carriers were compared to those from *HLA-C2* homozygotes and 3DL1^+^ cells from *Bw4* carriers were compared to 3DL1^+^ cells from *Bw6* homozygotes. There were no significant differences in the frequency of CD16^+^ cells in the educated and uneducated groups.

## Discussion

In this report we show that a lower frequency of CD56^dim^ NK cells expressing no NKG2A, 2DL1, 2DL3, or 3DL1 were CD16^+^ compared those expressing NKG2A alone, whereas a higher frequency of CD56^dim^ NKs expressing none of these iNKRs were CD16^+^ compared to those expressing the iKIR 2DL1, 2DL3, or 3DL1 alone. Whether 2DL1, 2DL3, or 3DL1^+^ NK cells developed in a host carrying alleles encoding HLA variants able to educate NK cells expressing these iKIR or not, did not affect the frequency of CD16^+^ cells in these NK cell populations.

CD16 is a low affinity activating Fc receptor (FcRIIIa) on NK cells, monocytes and neutrophils [39]. Its engagement on NK cells leads to the induction of several functions such as cytokine/chemokine secretion and expression of CD107a. Ab dependent NK cell activation also results in the delivery of GrB to target cells and their eventual cytolysis by ADCC. NK cell-mediated ADCC involves the recognition of a cognate antigen on target cells by the fragment variable (Fv) portion of an IgG Ab molecule and the ligation of the fragment crystallizable (Fc) region to CD16 on NK cells.

The higher frequencies of CD16^+^, CD16^+^NKG2A^+^, and CD16^+^CD57^+^ cells than their CD16^-^ counterparts observed in total CD56^+^ NK cells reflect the higher percentage of CD16^+^ cells among CD56^dim^ than CD56^bright^ NK cells in the periphery [32,33]. The higher proportion of CD16^-^ than CD16^+^ cells within the CD56^bright^ and NKG2A^+^CD56^bright^ populations is consistent with CD56^bright^ NKs being at an earlier stage of differentiation than CD56^dim^ NK cells [31,36,40]. As NK cells differentiate from CD56^bright^ to CD56^dim^, NKG2A expression declines and KIR expression increases [31,34,35]. CD57^+^ cells have also been reported to proliferate more poorly, express more CD16, and mediate more potent CD16 dependent cytotoxicity [36]. In support of this, we found that CD57^+^CD56^dim^ NKs were significantly more likely to co-express CD16.

That single iKIR^+^ NK cells are less likely to be CD16^+^ has not been reported previously. This may be partly due to the gating strategies and Ab panels that have been used to detect CD56, CD16, and iKIR on NK cells. Since NKG2A^+^ cells co-express CD16^+^ at a higher frequency than iNKR^-^ NK cells, if they are included in comparisons of NK cells positive versus negative for particular iKIR they may mask the differences in CD16 frequency in comparisons of single iKIR^+/-^ NK cell pairs. This may in part explain the results presented in Fig 2A where NKG2A^+^ cells were not excluded and NK cells expressing any combination of the iKIR screened for were gated on. Inclusion of CD56^bright^ NK cells in such analyses may also mask differences in CD16 expression on iKIR^+/-^ NK cell pairs. Although they only make up ~10% of the total CD56^+^ NK cell pool they are almost all NKG2A^+^ and rarely KIR^+^ and thus may skew results for the expression of CD16 on iKIR^+/-^ pairs within the CD56^dim^ compartment [35].

iKIR expression is required for NK cell education [1,3]. The finding that there are fewer CD16^+^ cells among iKIR^+^ compared to iKIR^-^ NK cells whether they are from subjects who express HLA ligands that would support the education of these NK cells would make iKIR^+^ NK cells poorer ADCC effector cells than their iKIR^-^ counterparts, as CD16 is crucial for ADCC activity. This could have contributed to the absence of an impact of educating 3DL1/Bw4 pairs on ADCC potency measured by assessing the frequency of the delivery of GrB to gp120 coated CEM, as previously reported [24]. Using the same data set as the one described in Isitman et al. [24] we stratified the results for %GrB^+^ CEM according to whether the effector cells used in the ADCC-GTL assay came from subjects positive or not for the educating 2DL1/HLA-C2 pair or the 2DL3/HLA-C1 pair. In neither case did we observe differences in the %GrB^+^ CEM cells when effector cells from subjects carrying educating versus non educating combinations were used (not shown).

It is important to note that not all possible iKIR were stained for, which is a limitation of this study. The reason we did not stain for KIR2DL2 is that there is no commercially available Ab that specifically detects this receptor without also detecting 2DL3 with or without KIR2DS2. Abs to other iKIR such as KIR2DL5, KIR3DL2, or KIR3DL3 were not included in the panel. The inhibitory function of these potential iKIR is not well characterized nor is their ligand specificity. In summary, it is possible that subsets of the cells staining as 2DL1^+^, 2DL3^+^, 3DL1^+^, NKG2A^+^, or iNKR^-^ may co-express these additional iKIR or activating KIR. At present, there is no information on the expression of CD16 on NK cell subsets expressing one or more of these unstained for iKIR.

Interest in ADCC increased when analyses of the results of the RV144 Thai trial revealed that IgG Abs to the V1/V2 low loop of the HIV Env, together with low levels of IgA and elevated levels of ADCC activity, may have contributed to the modest protection against HIV infection observed in this trial [41,42]. This, and the explosion of HIV-Env specific broadly neutralizing Abs, which also mediate ADCC has promoted interest in exploiting Fc-mediated effector functions for protection against HIV infection in a vaccine setting and for HIV therapy [43–47]. Our findings that NK cells that have the potential to be educated through iKIR are less likely to co-express CD16 suggests that non iKIR educated NK cells are the more likely mediators of ADCC. The implication of this observation is that vaccine or therapeutic strategies targeting NK cell mediated ADCC through the induction of ADCC-competent Abs will not be limited by inter-individual genetic differences that determine the potential potency of NK cell education.

## Supporting Information

S1 Fig The gating strategy used for detecting NK cells. The live lymphocytic singlet population was used to gate on NK cells, which were defined as CD3^-^CD56^+^ (CD56^total^), CD3^-^CD56^dim^, and CD3^-^CD56^bright^. SSC-A = side scatter area; FSC-H = forward scatter height; FSC-A = forward scatter area.
(TIF)

S1 Table Data used to create Fig 1B. Frequency of CD16^+/-^ cells among total CD56^+^, CD56^dim^ and CD56^bright^ NK cells.
(DOCX)

S2 Table Data used to create Fig 1C. Frequency of NKG2A+ cells among total CD56^+^, CD56^dim^ and CD56^bright^ NK cells.
(DOCX)

S3 Table Data used to create Fig 1D. Frequency of CD16^+/-^ NKG2A^+^ cells among total CD56^+^, CD56^dim^ and CD56^bright^ NK cells.
(DOCX)

S4 Table Data used to create Fig 1E. Frequency of CD57^+^ cells among total CD56^+^, CD56^dim^ and CD56^bright^ NK cells.
(DOCX)

S5 Table Data used to create Fig 1F. Frequency of CD16^+/-^ CD57^+^ cells among total CD56^+^, CD56^dim^ and CD56^bright^ NK cells.
(DOCX)

S6 Table Data used to create Fig 2A. Frequency of CD56^+^ NK cells among Killer Immunoglobulin-like Receptor (KIR)^+/-^CD16^+/-^ cells.
(DOCX)

S7 Table Data used to create Fig 2C. Frequency of CD16^+^ cells among CD56^dim^ NK cells expressing NKG2A, KIR2DL1 (2DL1), KIR2DL3 (2DL3) or KIR3DL1 (3DL1) to the exclusion of the other inhibitory NK receptors (iNKR) versus none of these iNKR.
(DOCX)

S8 Table Data used to create Fig 3. Frequency of CD56^dim^ CD16^+^ cells among educated and uneducated KIR2DL1 (2DL1)^+^, KIR2DL3 (2DL3)^+^ and KIR3DL1 (3DL1)^+^ NK cells.
(DOCX)
