# Supplementary figures and images for: NK Cells Expressing the Inhibitory Killer Immunoglobulin-Like Receptors (iKIR) KIR2DL1, KIR2DL3 and KIR3DL1 Are Less Likely to Be CD16+ than Their iKIR Negative Counterparts
Source: PLoS One. 2016 Oct 12;11(10):e0164517. doi: 10.1371/journal.pone.0164517 (PMC5061331; doi:10.1371/journal.pone.0164517)

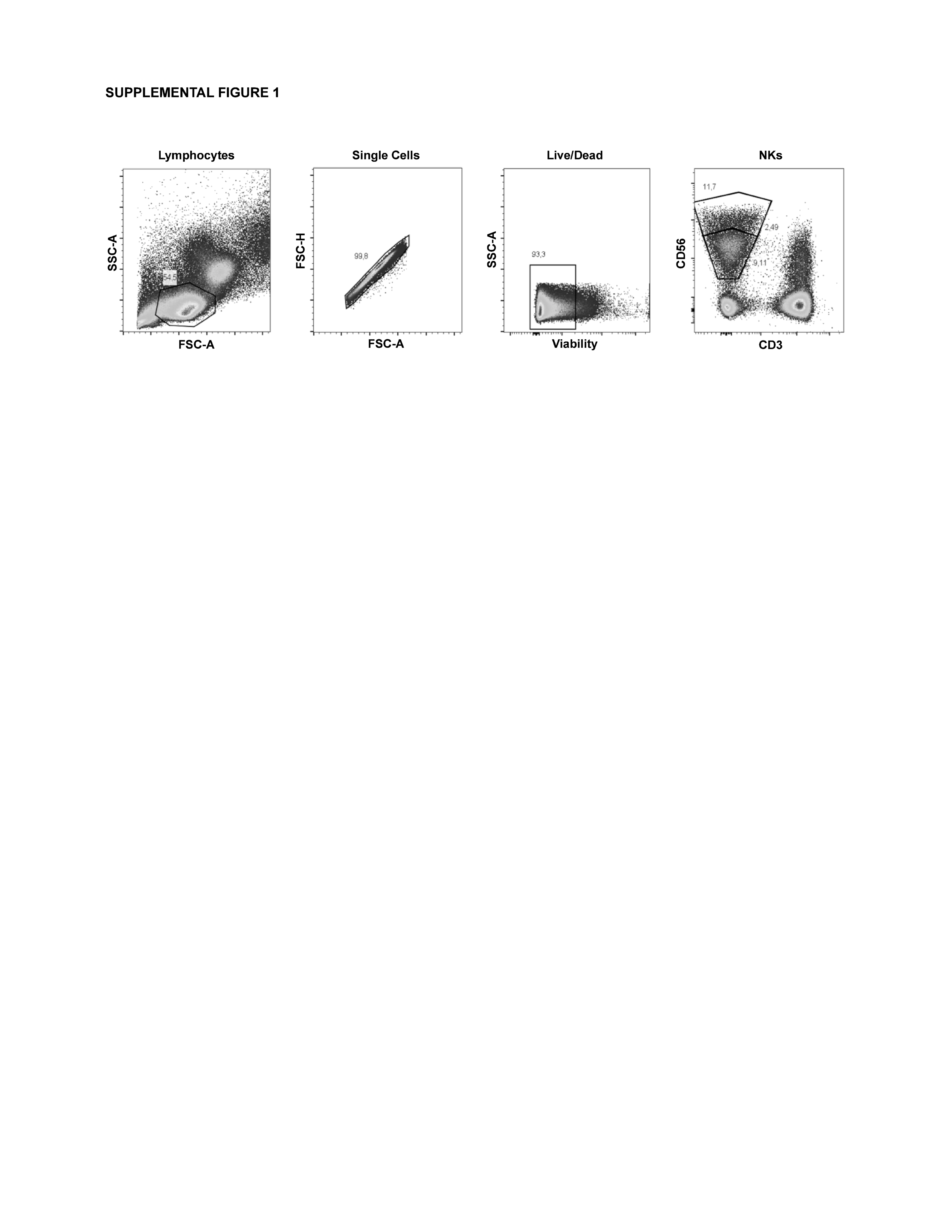

Supplement: S1 Fig — The live lymphocytic singlet population was used to gate on NK cells, which were defined as CD3-CD56+ (CD56total), CD3-CD56dim, and CD3-CD56bright. SSC-A = side scatter area; FSC-H = forward scatter height; FSC-A = forward scatter area. (TIF) [file pone.0164517.s001.TIF]
