# Supplementary material for: NK Cells Expressing the Inhibitory Killer Immunoglobulin-Like Receptors (iKIR) KIR2DL1, KIR2DL3 and KIR3DL1 Are Less Likely to Be CD16+ than Their iKIR Negative Counterparts
Source: PLoS One. 2016 Oct 12;11(10):e0164517. doi: 10.1371/journal.pone.0164517 (PMC5061331; doi:10.1371/journal.pone.0164517)
Supplement: S1 Table — Frequency of CD16+/- cells among total CD56+, CD56dim and CD56bright NK cells. (DOCX) [file pone.0164517.s002.docx]

| \| **S1 Table. Data used to create Fig 1B.** \| \| \| \|  \|  \|  \| \| --- \| --- \| --- \| --- \| --- \| --- \| --- \| \|  \| CD56^total^ \| \| CD56^dim^ \| \| CD56^bright^ \| \| \| Donor \| CD16^-^ \| CD16^+^ \| CD16^-^ \| CD16^+^ \| CD16^-^ \| CD16^+^ \| \| 1 \| 79.4 \| 20.6 \| 77.9 \| 22.1 \| 88.4 \| 11.6 \| \| 2 \| 69.9 \| 30.1 \| 59.4 \| 40.6 \| 63.3 \| 36.7 \| \| 3 \| 26.9 \| 73.1 \| 32.5 \| 67.5 \| 50.2 \| 49.8 \| \| 4 \| 72.1 \| 27.9 \| 71.5 \| 28.5 \| 89.9 \| 10.1 \| \| 5 \| 35.5 \| 64.5 \| 26.4 \| 73.6 \| 68.6 \| 31.4 \| \| 6 \| 27.3 \| 72.7 \| 21.8 \| 78.2 \| 50.8 \| 49.2 \| \| 7 \| 25.5 \| 74.5 \| 15.4 \| 84.6 \| 63.5 \| 36.5 \| \| 8 \| 19.1 \| 80.9 \| 16.3 \| 83.7 \| 81.2 \| 18.8 \| \| 9 \| 43.1 \| 56.9 \| 41.7 \| 58.3 \| 66.8 \| 33.2 \| \| 10 \| 36.8 \| 63.2 \| 28.6 \| 71.4 \| 62.3 \| 37.7 \| \| 11 \| 50.4 \| 49.6 \| 41 \| 59 \| 72.2 \| 27.8 \| \| 12 \| 64.9 \| 35.1 \| 64.3 \| 35.7 \| 91.3 \| 8.7 \| \| 13 \| 9.3 \| 90.7 \| 6.4 \| 93.6 \| 29.1 \| 70.9 \| \| 14 \| 11.3 \| 88.7 \| 8.4 \| 91.6 \| 43.8 \| 56.2 \| \| 15 \| 13 \| 87 \| 7.9 \| 92.1 \| 50.6 \| 49.4 \| \| 16 \| 25.8 \| 74.2 \| 14.7 \| 85.3 \| 56.5 \| 43.5 \| \| 17 \| 6.8 \| 93.2 \| 1.9 \| 98.1 \| 24 \| 76 \| \| 18 \| 18.2 \| 81.8 \| 15.9 \| 84.1 \| 35.75 \| 64.25 \| \| 19 \| 7.93 \| 92.1 \| 6.56 \| 93.4 \| 16.5 \| 83.5 \| \| 20 \| 51.8 \| 48.2 \| 32.4 \| 67.6 \| 81.7 \| 18.3 \| \| 21 \| 11.2 \| 88.8 \| 8.92 \| 91.1 \| 38.7 \| 61.3 \| \| 22 \| 8.75 \| 91.3 \| 5.28 \| 94.8 \| 45.6 \| 54.4 \| \| 23 \| 35.8 \| 64.2 \| 22.2 \| 77.8 \| 58.1 \| 41.9 \| \| 24 \| 14.4 \| 85.6 \| 9.74 \| 90.26 \| 49.6 \| 50.4 \| \| 25 \| 14.6 \| 85.4 \| 8.9 \| 91.1 \| 48.2 \| 51.8 \| \| 26 \| 45.5 \| 54.5 \| 42 \| 58 \| 37.8 \| 62.2 \| | | |  |  |  |
| --- | --- | --- | --- | --- | --- | --- | --- | --- | --- | --- | --- | --- | --- | --- | --- | --- | --- | --- | --- | --- | --- | --- | --- | --- | --- | --- | --- | --- | --- | --- | --- | --- | --- | --- | --- | --- | --- | --- | --- | --- | --- | --- | --- | --- | --- | --- | --- | --- | --- | --- | --- | --- | --- | --- | --- | --- | --- | --- | --- | --- | --- | --- | --- | --- | --- | --- | --- | --- | --- | --- | --- | --- | --- | --- | --- | --- | --- | --- | --- | --- | --- | --- | --- | --- | --- | --- | --- | --- | --- | --- | --- | --- | --- | --- | --- | --- | --- | --- | --- | --- | --- | --- | --- | --- | --- | --- | --- | --- | --- | --- | --- | --- | --- | --- | --- | --- | --- | --- | --- | --- | --- | --- | --- | --- | --- | --- | --- | --- | --- | --- | --- | --- | --- | --- | --- | --- | --- | --- | --- | --- | --- | --- | --- | --- | --- | --- | --- | --- | --- | --- | --- | --- | --- | --- | --- | --- | --- | --- | --- | --- | --- | --- | --- | --- | --- | --- | --- | --- | --- | --- | --- | --- | --- | --- | --- | --- | --- | --- | --- | --- | --- | --- | --- | --- | --- | --- | --- | --- | --- | --- | --- | --- | --- | --- | --- | --- | --- | --- | --- | --- | --- | --- | --- | --- | --- | --- | --- | --- |
|  |  |  | |  | |
