# Supplementary material for: NK Cells Expressing the Inhibitory Killer Immunoglobulin-Like Receptors (iKIR) KIR2DL1, KIR2DL3 and KIR3DL1 Are Less Likely to Be CD16+ than Their iKIR Negative Counterparts
Source: PLoS One. 2016 Oct 12;11(10):e0164517. doi: 10.1371/journal.pone.0164517 (PMC5061331; doi:10.1371/journal.pone.0164517)
Supplement: S2 Table — Frequency of NKG2A+ cells among total CD56+, CD56dim and CD56bright NK cells. (DOCX) [file pone.0164517.s003.docx]

| **S2 Table. Data used to create Fig 1C.** | | | | |
| --- | --- | --- | --- | --- |
|  |  | | |  |
| Donor | CD56^total^ | CD56^dim^ | CD56^bright^ |  |
| 1 | 72.3 | 68.8 | 95.9 |  |
| 2 | 71.3 | 70.7 | 93 |  |
| 3 | 53.8 | 46.5 | 89.8 |  |
| 4 | 27 | 25 | 93.3 |  |
| 5 | 76.5 | 71 | 96.3 |  |
| 6 | 57.7 | 56.8 | 95.3 |  |
| 7 | 75.7 | 70.6 | 96.9 |  |
| 8 | 76.8 | 76.2 | 93 |  |
| 9 | 64.5 | 63.7 | 95.1 |  |
| 10 | 76.5 | 70.2 | 97.4 |  |
| 11 | 58.3 | 42.1 | 97.8 |  |
| 12 | 93.5 | 55.5 | 93.5 |  |
| 13 | 65.5 | 61.8 | 91.3 |  |
| 14 | 48.7 | 45.7 | 85.8 |  |
| 15 | 56.2 | 51.8 | 89.1 |  |
| 16 | 56 | 43 | 92.8 |  |
| 17 | 28.1 | 15.1 | 73.3 |  |
| 18 | 54.9 | 49.35 | 97.25 |  |
| 19 | 53.5 | 50.8 | 80.3 |  |
| 20 | 79.9 | 79.6 | 80.6 |  |
| 21 | 50.2 | 48.4 | 71.3 |  |
| 22 | 38.3 | 35.6 | 70.2 |  |
| 23 | 64.8 | 64 | 92 |  |
| 24 | 55 | 51.4 | 83.5 |  |
| 25 | 36.6 | 30.9 | 71.5 |  |
| 26 | 64.3 | 62.8 | 85 |  |
