# Supplementary material for: NK Cells Expressing the Inhibitory Killer Immunoglobulin-Like Receptors (iKIR) KIR2DL1, KIR2DL3 and KIR3DL1 Are Less Likely to Be CD16+ than Their iKIR Negative Counterparts
Source: PLoS One. 2016 Oct 12;11(10):e0164517. doi: 10.1371/journal.pone.0164517 (PMC5061331; doi:10.1371/journal.pone.0164517)
Supplement: S3 Table — Frequency of CD16+/- NKG2A+ cells among total CD56+, CD56dim and CD56bright NK cells. (DOCX) [file pone.0164517.s004.docx]

| **S3 Table. Data used to create Fig 1D.** | | | | | |  |
| --- | --- | --- | --- | --- | --- | --- |
|  | CD56^total^ | | CD56^dim^ | | CD56^bright^ | |
| Donor | CD16^-^ | CD16^+^ | CD16^-^ | CD16^+^ | CD16^-^ | CD16^+^ |
| 1 | 77.8 | 22.2 | 75.4 | 24.6 | 88.6 | 11.4 |
| 2 | 61.9 | 38.1 | 61.9 | 38.1 | 62.5 | 37.5 |
| 3 | 21.4 | 78.6 | 10 | 90 | 49.9 | 50.1 |
| 4 | 61.6 | 38.4 | 58.6 | 41.4 | 89.4 | 10.6 |
| 5 | 38.6 | 61.4 | 26.8 | 73.2 | 70 | 30 |
| 6 | 19.8 | 80.2 | 15 | 85 | 49.6 | 50.4 |
| 7 | 25.8 | 74.2 | 12.4 | 87.6 | 64.1 | 35.9 |
| 8 | 18.5 | 81.5 | 15.2 | 84.8 | 82.2 | 17.8 |
| 9 | 40.6 | 59.4 | 39 | 61 | 66.7 | 33.3 |
| 10 | 35.3 | 64.7 | 23.5 | 76.5 | 62.2 | 37.8 |
| 11 | 59.5 | 40.5 | 47.4 | 52.6 | 72.3 | 27.7 |
| 12 | 90.7 | 9.3 | 63.8 | 36.2 | 90.7 | 9.3 |
| 13 | 11.6 | 88.4 | 6.7 | 93.3 | 31.3 | 68.7 |
| 14 | 14.7 | 85.3 | 9 | 91 | 44.4 | 55.6 |
| 15 | 17.4 | 82.6 | 8.1 | 91.9 | 51.5 | 48.5 |
| 16 | 34.4 | 65.6 | 15.1 | 84.9 | 56.5 | 43.5 |
| 17 | 18.7 | 81.3 | 5 | 95 | 28.5 | 71.5 |
| 18 | 22.75 | 77.25 | 19.1 | 80.9 | 35.95 | 64.05 |
| 19 | 5.9 | 94.1 | 3.8 | 96.2 | 17.1 | 82.9 |
| 20 | 48.3 | 51.7 | 26.5 | 73.5 | 81.3 | 18.7 |
| 21 | 11.4 | 88.6 | 6.9 | 93.1 | 48.9 | 51.1 |
| 22 | 12.4 | 87.6 | 4.9 | 95.1 | 54.4 | 45.6 |
| 23 | 40.4 | 59.6 | 12.9 | 87.1 | 58.6 | 41.4 |
| 24 | 17.7 | 82.3 | 9.9 | 90.1 | 54.1 | 45.9 |
| 25 | 24.4 | 75.6 | 9.6 | 90.4 | 63.3 | 36.7 |
| 26 | 47.2 | 52.8 | 43.3 | 56.7 | 48.4 | 51.6 |
