# Supplementary material for: NK Cells Expressing the Inhibitory Killer Immunoglobulin-Like Receptors (iKIR) KIR2DL1, KIR2DL3 and KIR3DL1 Are Less Likely to Be CD16+ than Their iKIR Negative Counterparts
Source: PLoS One. 2016 Oct 12;11(10):e0164517. doi: 10.1371/journal.pone.0164517 (PMC5061331; doi:10.1371/journal.pone.0164517)
Supplement: S4 Table — Frequency of CD57+ cells among total CD56+, CD56dim and CD56bright NK cells. (DOCX) [file pone.0164517.s005.docx]

| **S4 Table. Data used to create Fig 1E.** | | | |
| --- | --- | --- | --- |
|  |  | |  |
| Donor | CD56^total^ | CD56^dim^ | CD56^bright^ |
| 1 | 38.6 | 44.1 | 4.65 |
| 2 | 36.1 | 36.5 | 30.2 |
| 3 | 28.9 | 34.1 | 4.78 |
| 4 | 73.1 | 75.4 | 4.93 |
| 5 | 50.5 | 62.6 | 7.06 |
| 6 | 18.7 | 21.2 | 0 |
| 7 | 32.7 | 40.2 | 3.74 |
| 8 | 50.2 | 52.3 | 1.26 |
| 9 | 53 | 55.1 | 4.64 |
| 10 | 21 | 26.6 | 3.65 |
| 11 | 33.3 | 46.5 | 1.83 |
| 12 | 2.17 | 51.9 | 2.17 |
| 13 | 50 | 55.1 | 14.7 |
| 14 | 51.6 | 55.4 | 8.74 |
| 15 | 33.3 | 37.3 | 3.92 |
| 16 | 24 | 32.2 | 1.08 |
| 17 | 57.5 | 70 | 14.3 |
| 18 | 36.9 | 42.25 | 0.52 |
| 19 | 80.8 | 83.7 | 53.7 |
| 20 | 43.5 | 66.2 | 8.6 |
| 21 | 79.5 | 82.6 | 41.8 |
| 22 | 58.5 | 61.5 | 24.5 |
| 23 | 31.6 | 46.2 | 6.86 |
| 24 | 30.4 | 32.3 | 16.7 |
| 25 | 50.3 | 54.6 | 24.9 |
| 26 | 60 | 62.1 | 34.4 |
