# Supplementary material for: NK Cells Expressing the Inhibitory Killer Immunoglobulin-Like Receptors (iKIR) KIR2DL1, KIR2DL3 and KIR3DL1 Are Less Likely to Be CD16+ than Their iKIR Negative Counterparts
Source: PLoS One. 2016 Oct 12;11(10):e0164517. doi: 10.1371/journal.pone.0164517 (PMC5061331; doi:10.1371/journal.pone.0164517)
Supplement: S5 Table — Frequency of CD16+/- CD57+ cells among total CD56+, CD56dim and CD56bright NK cells. (DOCX) [file pone.0164517.s006.docx]

| **S5 Table. Data used to create Fig 1F.** | | | | | |  |
| --- | --- | --- | --- | --- | --- | --- |
|  | CD56^total^ | | CD56^dim^ | | CD56^bright^ | |
| Donor | CD16^-^ | CD16^+^ | CD16^-^ | CD16^+^ | CD16^-^ | CD16^+^ |
| 1 | 78.5 | 21.5 | 78.6 | 21.4 | 75.7 | 24.3 |
| 2 | 50.4 | 49.6 | 50.5 | 49.5 | 48 | 52 |
| 3 | 4.7 | 95.3 | 4.5 | 95.5 | 6.2 | 93.8 |
| 4 | 71.6 | 28.4 | 71.6 | 28.4 | 95 | 5 |
| 5 | 18.4 | 81.6 | 18.7 | 81.3 | 8.4 | 91.6 |
| 6 | 18.6 | 81.4 | 18.2 | 81.8 | 100 | 0 |
| 7 | 8.1 | 91.9 | 8.3 | 91.7 | 1.5 | 98.5 |
| 8 | 6.6 | 93.4 | 6.5 | 93.5 | 20 | 80 |
| 9 | 33.1 | 66.9 | 32.7 | 67.3 | 38.9 | 61.1 |
| 10 | 23.1 | 76.9 | 22.5 | 77.5 | 34.7 | 65.3 |
| 11 | 22.9 | 77.1 | 21.3 | 78.7 | 100 | 0 |
| 12 | 100 | 0 | 57.3 | 42.7 | 100 | 0 |
| 13 | 3.4 | 96.6 | 3.5 | 96.5 | 29.1 | 70.9 |
| 14 | 4.7 | 95.3 | 4.7 | 95.3 | 3.1 | 96.9 |
| 15 | 3.4 | 96.6 | 3.3 | 96.7 | 4.3 | 95.7 |
| 16 | 1.8 | 98.2 | 1.3 | 98.7 | 28.6 | 71.4 |
| 17 | 0.4 | 99.6 | 0.3 | 99.7 | 0.8 | 99.2 |
| 18 | 10 | 90 | 10.2 | 89.8 | 0 | 100 |
| 19 | 5.5 | 94.5 | 5.2 | 94.8 | 3.4 | 96.6 |
| 20 | 15.8 | 84.2 | 11.7 | 88.3 | 62.5 | 37.5 |
| 21 | 7.5 | 92.5 | 7.7 | 92.3 | 2 | 98 |
| 22 | 3.5 | 96.5 | 3.3 | 96.7 | 2.4 | 97.6 |
| 23 | 20.4 | 79.6 | 18.4 | 81.6 | 41.3 | 58.7 |
| 24 | 7.6 | 92.4 | 7.4 | 92.6 | 9.7 | 90.3 |
| 25 | 6.7 | 93.3 | 6.2 | 93.8 | 10.2 | 89.8 |
| 26 | 40.8 | 59.2 | 37.3 | 62.7 | 22.6 | 77.4 |
