# Supplementary material for: NK Cells Expressing the Inhibitory Killer Immunoglobulin-Like Receptors (iKIR) KIR2DL1, KIR2DL3 and KIR3DL1 Are Less Likely to Be CD16+ than Their iKIR Negative Counterparts
Source: PLoS One. 2016 Oct 12;11(10):e0164517. doi: 10.1371/journal.pone.0164517 (PMC5061331; doi:10.1371/journal.pone.0164517)
Supplement: S6 Table — Frequency of CD56+ NK cells among Killer Immunoglobulin-like Receptor (KIR)+/-CD16+/- cells. (DOCX) [file pone.0164517.s007.docx]

| **S6 Table. Data used to create Fig 2A.** | | | | |
| --- | --- | --- | --- | --- |
|  | CD16- | | CD16+ | |
| Donor | KIR^-^ | KIR^+^ | KIR^-^ | KIR^+^ |
| 1 | 66.62 | 33.38 | 64.43 | 35.57 |
| 2 | 80.89 | 19.11 | 72.89 | 27.11 |
| 3 | 76.83 | 23.17 | 45.7 | 54.3 |
| 4 | 72.48 | 27.52 | 73.41 | 26.59 |
| 5 | 59.11 | 40.89 | 36.83 | 63.17 |
| 6 | 74.43 | 25.57 | 43.34 | 56.66 |
| 7 | 74.62 | 25.38 | 53.5 | 46.5 |
| 8 | 76.9 | 23.1 | 64.97 | 35.03 |
| 9 | 41.53 | 58.47 | 38.47 | 61.53 |
| 10 | 73.57 | 26.43 | 71.57 | 28.43 |
| 11 | 73.99 | 26.01 | 47.56 | 52.44 |
| 12 | 61 | 39 | 54.77 | 45.23 |
| 13 | 76.88 | 23.12 | 58.37 | 41.63 |
| 14 | 60.32 | 39.68 | 33.91 | 66.09 |
| 15 | 56.49 | 43.51 | 36.19 | 63.81 |
| 16 | 75.36 | 24.64 | 61.52 | 38.48 |
| 17 | 17.98 | 82.02 | 64.87 | 35.13 |
| 18 | 33.59 | 66.41 | 30.92 | 69.08 |
| 19 | 50.69 | 49.31 | 41.82 | 58.18 |
| 20 | 32.65 | 67.35 | 24.42 | 75.58 |
| 21 | 37.11 | 62.29 | 27.89 | 72.1 |
| 22 | 26.02 | 73.98 | 23.58 | 76.42 |
| 23 | 73 | 27 | 40.47 | 59.53 |
| 24 | 74.5 | 25.5 | 37.73 | 62.27 |
| 25 | 74.5 | 25.5 | 38.7 | 61.3 |
| 26 | 71.9 | 28.1 | 48.4 | 51.6 |
