# Supplementary material for: NK Cells Expressing the Inhibitory Killer Immunoglobulin-Like Receptors (iKIR) KIR2DL1, KIR2DL3 and KIR3DL1 Are Less Likely to Be CD16+ than Their iKIR Negative Counterparts
Source: PLoS One. 2016 Oct 12;11(10):e0164517. doi: 10.1371/journal.pone.0164517 (PMC5061331; doi:10.1371/journal.pone.0164517)
Supplement: S7 Table — Frequency of CD16+ cells among CD56dim NK cells expressing NKG2A, KIR2DL1 (2DL1), KIR2DL3 (2DL3) or KIR3DL1 (3DL1) to the exclusion of the other inhibitory NK receptors (iNKR) versus none of these iNKR. (DOCX) [file pone.0164517.s008.docx]

| **S7 Table. Data used to create Fig 2C.** | | | | | |
| --- | --- | --- | --- | --- | --- |
|  | CD16+ | | | | |
| Donor | NKG2A^+^ | 2DL1^+^ | 2DL3^+^ | 3DL1^+^ | iNKR^-^ |
| 1 | 10.3 | 0.89 | 0.44 | 0.79 | 2.44 |
| 2 | 19.9 |  | 2.25 | 0.99 | 9.64 |
| 3 | 22 | 3.72 |  | 16.1 | 14 |
| 4 | 6.57 | 1.19 | 3.07 | 0.59 | 12.5 |
| 5 | 21.2 | 2.3 | 4.41 | 0.99 | 2.7 |
| 6 | 21 | 7.1 | 8.83 |  | 7.06 |
| 7 | 31.5 | 5.69 | 2.81 | 1.8 | 6.46 |
| 8 | 43.8 | 1.89 | 2.92 | 1.93 | 10.6 |
| 9 | 18.4 | 1.14 | 3.18 | 4.57 | 18.4 |
| 10 | 39.2 | 2.74 |  | 2.58 | 11.9 |
| 11 | 12.2 | 4.33 | 14.4 |  | 16.4 |
| 12 | 13.2 | 0.77 | 4.56 | 1.84 | 6.19 |
| 13 | 40.9 | 4.04 | 6.62 | 3.84 | 13.7 |
| 14 | 18.8 | 4.44 | 1.25 | 16.2 | 12.3 |
| 15 | 23.8 | 1.7 | 10.5 | 6.7 | 9.56 |
| 16 | 28.3 | 6.06 | 5.31 | 6.23 | 24.1 |
| 17 | 3.37 | 3.07 | 29.8 | 5.7 | 12.8 |
| 18 | 13.07 | 2.5 | 5.22 | 7.03 | 3.075 |
| 19 | 33.5 | 1.89 | 8.32 | 8.77 | 20.8 |
| 20 | 46.8 | 0.7 | 1.76 |  | 5.99 |
| 21 | 37.8 | 17.5 |  | 0.13 | 28.1 |
| 22 | 25.6 | 1.5 | 8.15 | 6.55 | 42.8 |
| 23 | 18.4 | 6.73 | 2.88 | 1.32 | 6.05 |
| 24 | 18.2 | 13.3 | 2.84 | 4.5 | 13.3 |
| 25 | 14.6 | 10.5 | 6.76 | 15.7 | 17.5 |
| 26 | 21.7 | 5.29 | 2.27 | 2.36 | 6.37 |
