# Supplementary material for: NK Cells Expressing the Inhibitory Killer Immunoglobulin-Like Receptors (iKIR) KIR2DL1, KIR2DL3 and KIR3DL1 Are Less Likely to Be CD16+ than Their iKIR Negative Counterparts
Source: PLoS One. 2016 Oct 12;11(10):e0164517. doi: 10.1371/journal.pone.0164517 (PMC5061331; doi:10.1371/journal.pone.0164517)
Supplement: S8 Table — Frequency of CD56dim CD16+ cells among educated and uneducated KIR2DL1 (2DL1)+, KIR2DL3 (2DL3)+ and KIR3DL1 (3DL1)+ NK cells. (DOCX) [file pone.0164517.s009.docx]

| **S8 Table. Data used to create Fig 3.** | | | | | |  |  |  |  |  |  |
| --- | --- | --- | --- | --- | --- | --- | --- | --- | --- | --- | --- |
|  | Education^+^ |  | Education^-^ |  | Education^+^ |  | Education^-^ |  | Education^+^ |  | Education^-^ |
|  | 2DL1+ |  | 2DL1- |  | 2DL3+ |  | 2DL3- |  | 3DL1+ |  | 3DL1- |
| Donor | HLA-C2+ | Donor | HLA-C2- | Donor | HLA-C1+ | Donor | HLA-C1- | Donor | HLA-Bw4+ | Donor | HLA-Bw4- |
| 11 | 4.33 | 6 | 7.1 | 6 | 8.83 | 11 | 14.4 | 1 | 0.79 | 13 | 3.84 |
| 3 | 3.72 | 10 | 2.74 | 12 | 4.56 | 8 | 2.92 | 4 | 0.59 | 14 | 16.2 |
| 8 | 1.89 | 12 | 0.77 | 1 | 0.44 | 7 | 2.81 | 5 | 0.99 | 15 | 6.7 |
| 7 | 5.69 | 1 | 0.89 | 4 | 3.07 | 2 | 2.25 | 7 | 1.8 | 16 | 6.23 |
| 19 | 1.89 | 4 | 1.19 | 5 | 4.41 | 19 | 8.32 | 2 | 0.99 | 23 | 1.32 |
| 20 | 0.7 | 5 | 2.3 | 9 | 3.18 | 20 | 1.76 | 9 | 4.57 | 24 | 4.5 |
| 15 | 17.5 | 9 | 1.14 | 13 | 6.62 | 22 | 8.15 | 17 | 5.7 | 25 | 15.7 |
| 22 | 1.5 | 13 | 4.04 | 14 | 1.25 |  |  | 18 | 7.03 | 26 | 2.36 |
|  |  | 14 | 4.44 | 23 | 2.84 |  |  |  |  |  |  |
|  |  | 24 | 13.3 | 26 | 6.76 |  |  |  |  |  |  |
|  |  | 25 | 10.5 |  |  |  |  |  |  |  |  |
|  |  |  |  |  |  |  |  |  |  |  |  |
